# Network analysis reveals a major role for 14q32 cluster miRNAs in determining transcriptional differences between IGHV-mutated and unmutated CLL

**DOI:** 10.1038/s41375-023-01918-9

**Published:** 2023-05-11

**Authors:** Dean Bryant, Lindsay Smith, Karly Rai Rogers-Broadway, Laura Karydis, Jeongmin Woo, Matthew D. Blunt, Francesco Forconi, Freda K. Stevenson, Christopher Goodnow, Amanda Russell, Peter Humburg, Graham Packham, Andrew J. Steele, Jonathan C. Strefford

**Affiliations:** 1 School of Cancer Sciences, Faculty of Medicine, University of Southampton, Southampton, UK; 2 School of Clinical and Experimental Sciences, Faculty of Medicine, University of Southampton, Southampton, UK; 3 Garvan Institute of Medical Research, Darlinghurst, Sydney, NSW 2010 Australia; 4 Cellular Genomics Futures Institute, UNSW Sydney, Sydney, NSW Australia

**Keywords:** Cancer genomics, Chronic lymphocytic leukaemia, Cancer epigenetics

## Abstract

Chronic lymphocytic leukaemia (CLL) cells can express unmutated (U-CLL) or mutated (M-CLL) immunoglobulin heavy chain (IGHV) genes with differing clinical behaviours, variable B cell receptor (BCR) signalling capacity and distinct transcriptional profiles. As it remains unclear how these differences reflect the tumour cells’ innate pre/post germinal centre origin or their BCR signalling competence, we applied mRNA/miRNA sequencing to 38 CLL cases categorised into three subsets by IGHV mutational status and BCR signalling capacity. We identified 492 mRNAs and 38 miRNAs differentially expressed between U-CLL and M-CLL, but only 9 mRNAs and 0 miRNAs associated with BCR competence within M-CLL. Of the IGHV-associated miRNAs, (14/38 (37%)) derived from chr14q32 clusters where all miRNAs were co-expressed with the *MEG3* lncRNA from a cancer associated imprinted locus. Integrative analysis of miRNA/mRNA data revealed pronounced regulatory potential for the 14q32 miRNAs, potentially accounting for up to 25% of the IGHV-related transcriptome signature. *GAB1*, a positive regulator of BCR signalling, was potentially regulated by five 14q32 miRNAs and we confirmed that two of these (miR-409-3p and miR-411-3p) significantly repressed activity of the *GAB1* 3′UTR. Our analysis demonstrates a potential key role of the 14q32 miRNA locus in the regulation of CLL-related gene regulation.

## Introduction

Chronic lymphocytic leukaemia (CLL) is a heterogeneous neoplasm of mature B-cell origin. Early genetic studies of the immunoglobulin heavy-chain variable region (IGHV), that encodes part of the B-cell receptor (BCR), identified two disease subtypes based on the degree of somatic hypermutation (SH) of the IGHV locus whereby CLL with mutated IGHV genes (M-CLL) and unmutated IGHV genes (U-CLL) typically have a favourable and poorer prognosis, respectively [1, 2]. Subsequent gene expression, DNA methylation and chromatin accessibility studies support a distinct cell of origin for these two CLL subtypes, with M-CLL and U-CLL exhibiting the expression profile of post-germinal centre memory cell, and pre-germinal centre B-cell, respectively [3, 4]. In addition, DNA methylation profiling further identified a third minor subgroup exhibiting an intermediate methylome between M-CLL and U-CLL, with distinct immunogenetic and genomic features and intermediate clinical outcome [5].

The B-cell receptor (BCR), as evidenced by the efficacy of BTK inhibition, is of critical importance to the progression of CLL [6]. CLL cells typically express immunoglobulin D (IgD) and M (IgM) on their cell surface, although at reduced levels compared to normal B cells and CLL is hypothesised to undergo BCR engagement by autoantigen [7]. Within this low range, tumour cells from CLL patients exhibit variation in both the level of surface IgM (sIgM) and in their capacity to signal via anti-IgM engagement. Whilst U-CLL typically exhibit higher sIgM levels and signalling capacity than M-CLL, there is overlap and higher functional levels of sIgM are associated with patient survival independent of IGHV status (median survival of 32, 81 and 183 months for signalling competent U-CLL, signalling competent M-CLL and signalling deficient M-CLL respectively [8]). The outcome of BCR signalling ranges from B cell activation to anergy, with possibly more of the latter in M-CLL [9]. However, how the differential responses to anti-IgM stimulation of the BCR operate in vivo is not yet fully understood.

MicroRNAs (miRNAs) are a class of short, ~22nt non-coding RNAs with important roles in the regulation of gene expression. Mapping miRNA *loci* at the genome level shows that miRNAs are often intragenic, located in introns, occur within clusters of co-regulated miRNAs and are expressed as a consequence of host gene transcription [10]. Most miRNAs are transcribed from DNA into primary miRNA prior to further processing in to precursor and mature species (reviewed in O’Brien et al. [11]). Analysis of 13q14 deletion in CLL was the first reported link between somatic genomic lesions and deregulation of miRNAs, specifically miR-15a and miR-16-1, negative regulators of the antiapoptotic proteins Bcl-2 and Mcl-1 [12, 13]. miR-34b/c, miR-21, miR-29, miR-125b, miR-181b, miR-17/92, miR-150, and miR-155 family miRNAs are also suggested to be biologically and clinically important in CLL, are differentially expressed compared to the normal B cells [14] and can be utilised to risk-stratify CLL patients into prognostically relevant subtypes [15]. Several miRNAs have been causally linked to CLL pathogenesis, including miR-15a/16-1, miR-29b/miR-181b, miR-181b and miR-34a that interact with the anti-apoptotic and cell cycle control genes *BCL2*, *TCL1*, *MCL1* and *TP53* respectively [16–19]. miR-150 and miR-155 are the most abundantly expressed miRNAs in CLL, where they regulate BCR signalling by targeting *FOXP1* and *GAB1* mRNAs and via downmodulation of the BCR phosphatase *SHIP1*, respectively [20, 21]. However, there remains uncertainty as to what degree the abnormal miRNAs reflect the cause or consequence of disrupted BCR signalling.

Whilst considerable research has described the biological basis underpinning the clinical heterogeneity observed in M-CLL and U-CLL, it remains unclear to what extent these differing biological features reflect the tumour cells innate pre/post-germinal centre origin or their acquired BCR signalling competence. Consequently, we performed a detailed multi-omics analysis of CLL samples differentiated by IGHV mutational status and BCR signalling capacity. By employing genome-wide miRNA and mRNA sequencing, with integration of matched DNA methylation, copy number analysis and sophisticated data integration approaches, we identify differential expression of a cluster of miRNAs within the *DLK1*-*DIO3* imprinted locus at 14q32, that associated with IGHV mutation status. Furthermore, we demonstrate a broad impact for the 14q32 miRNAs on the CLL transcriptome and identify the BCR regulating gene, *GAB1*, as a potential target of multiple 14q32 miRNAs.

## Methods

### Patient cohorts and sample characteristics

**Fig. 1:**
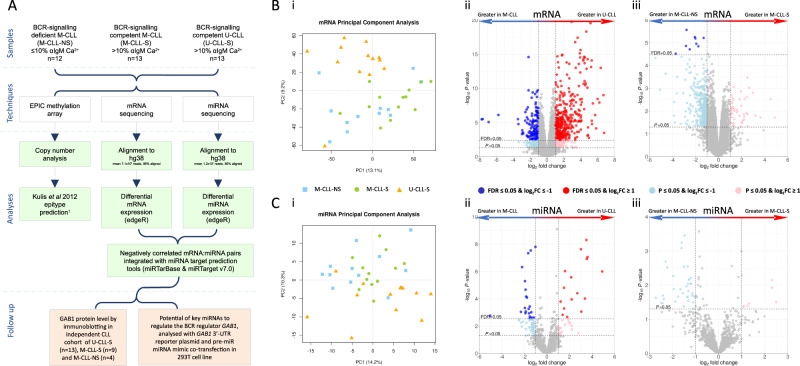
Project outline, PCA plots and volcano plots for pairwise differential expression comparisons. **A** Consort diagram showing the order of investigation of the mRNA, miRNA and DNA methylation profiles of CLL. **B** (i) PCA plots using mRNA data and volcano plots for mRNA pairwise comparisons of (ii) U-CLL vs. M-CLL and (iii) M-CLL-S vs. M-CLL-NS. **C** (i) PCA plots using miRNA data and volcano plots for miRNA pairwise comparisons of (ii) U-CLL vs. M-CLL and (iii) M-CLL-S vs. M-CLL-NS. Points represent a mRNA or miRNA. Dark blue/red are differentially expressed at FDR ≤ 0.05 and log_2_FC ≥ ±1 after correcting for multiple testing (BH), light blue/pink are significant at *P* ≤ 0.05 and log_2_FC ≥ ±1.

An overview of the cohort and methods applied in this study are depicted in Fig. 1A. Thirty-eight basal tumour samples, obtained from CLL patients diagnosed using the iwCLL guidelines [22] and managed at Southampton General Hospital (Southampton, UK), were selected based on IGHV mutational status using established cut-offs [1, 2] (Fig. S1A), and BCR signalling capacity with >10%/≤10% anti-IgM-induced Ca^2+^ mobilisation thresholds (Fig. S1B) determined as described previously [8]. Informed consent was obtained in accordance with the declaration of Helsinki and the study was approved by our regional research ethics committee. We used high purity (mean 89.5%, range 77–99%) (Fig. S1C), non-purified CLL cells for the initial transcriptome and miRNA sequencing and purified cells (Miltenyi B-CLL Isolation kit) for DNA methylation analysis and confirmatory miRNA sequencing. CLL cases were divided into three subgroups of BCR-signalling competent U-CLL (U-CLL-S, *n* = 13) and M-CLL (M-CLL-S, *n* = 13) and BCR-signalling deficient M-CLL (M-CLL-NS, *n* = 12). The absence of prior studies using similar technologies made power estimation difficult, instead, all available M-CLL-NS cases were used with comparable numbers of M-CLL-S and U-CLL-S to ensure maximum power for the analysis. Due to sample material limitations, GAB1 protein immunoblotting was performed on a subset of our main cohort comprising U-CLL-S (*n* = 5), M-CLL-S (*n* = 6) and M-CLL-NS (*n* = 4), and additional samples including U-CLL-S (*n* = 8) and M-CLL-S (*n* = 3).

A more comprehensive overview of subsequent methods is detailed in Supplementary Materials.

### mRNA and miRNA sequencing

mRNA sequencing was performed on RNA extracted with Qiagen RNeasy mini kits (Qiagen, Hilden, Germany). Libraries were prepared using TruSeq RNA kits (Illumina, Hayward, CA, USA) and sequenced using Illumina HiSeq2500 (*n* = 17) and HiSeq4000 (*n* = 21). Raw data was aligned to the hg38 reference genome using HISAT2 and read counts were calculated using HTseq-count against the Ensembl GRCh38.v94 GTF.

miRNA sequencing was performed on total RNA extracted with Qiagen miRNeasy kits. Libraries were prepared using Illumina TruSeq small RNA library kits and sequenced using Illumina HiSeq4000 (*n* = 17) and an Illumina HiSeq2500 (*n* = 21). Confirmatory miRNA sequencing to preclude the contribution of contaminating T-cell/monocytes was processed as before, but from purified CLL cells (Miltenyi B-CLL Isolation kit (Miltenyi, Bergisch Gladbach, Germany)), and sequenced on an Illumina HiSeq 4000. miRNA data in the form of fastq were aligned to the hg38 reference genome using BWA v0.7.12 (RRID:SCR_010910) and read counts calculated using HTseq-Count against miRbase v21.

### Data analysis

Differential gene expression analysis was performed in using EdgeR v3.32.1 (RRID:SCR_012802) for IGHV mutation status and/or CLL subgroup. miRNA:mRNA interaction analysis was performed using the R package miRComb with additional miRNA targets databases and plotted in CytoScape v3 (RRID:SCR_003032) to show negatively correlated miRNA:mRNA pairs present in miRNA target databases. Statistical analysis of miRNA:mRNA interaction enrichment was performed by comparing our network against 50,000 samples of randomly selected miRNAs/mRNAs using a one-way student’s *t*-test. All analyses were performed in R v.3.6.1 (RRID:SCR_001905). For statistical analysis of single miRNA, mRNA, immunoblot and luciferase level pairwise comparisons, Wilcoxon signed rank tests were used.

### EPIC DNA methylation array

DNA methylation was assessed in DNA extracted using QIAamp DNA Blood mini kits (Qiagen, Hilden, Germany), using Illumina EPIC DNA methylation arrays. Data processing and differential methylation analysis was performed using RnBeads v2.93 (RRID:SCR_010958). Conumee (Bioconductor) was used to produce copy number profiles (Tables S1 and S2), DNA methylation epitypes were determined using the process described previously [4].

### Immunoblotting

SDS-PAGE was performed on 3 × 10^6^ lysed CLL cells and run with equal protein loading on 10% Nu-PAGE Bis-Tris gel (Invitrogen, Waltham, MA, USA) with MOPS buffer (Invitrogen, Waltham, MA, USA). Protein was quantified using the Bio-Rad Protein Assay (Bio-Rad Laboratories Inc, Hercules, CA, USA.) and the blots stained with the following primary antibodies; rabbit anti-GAB (Cell Signaling Technology Europe B.V., Leiden, Netherlands, Cat no. 3232S) and mouse anti-Hsc70 (Insight Biotechnology Ltd., Wembley, UK, Cat no. sc-7298). Secondary antibodies were horseradish peroxidase-conjugated anti-rabbit/anti-mouse (Agilent Technologies LDA UK Limited, Cheshire, UK, Cat nos. P0448/P0447). Images were captured using the ChemiDoc-It Imaging System and quantified using ImageJ (RRID:SCR_003070). Representative immunoblots are shown in Supplementary Fig. 3 (Fig. S4).

### miRNA transfections

miRNA activity was quantified by co-transfecting 293T cells (ThermoFisher, Leicestershire, UK) with Lipofectamine 2000 (ThermoFisher, Leicestershire, UK), a human *GAB1* 3′-UTR reporter plasmid (containing 3770 bp immediately downstream of the end of the *GAB1* ORF cloned into pMirTarget, Origene), a control Renilla luciferase plasmid (Promega) and pre-miR miRNA mimics or pre-miR control 1 (ThermoFisher, Leicestershire, UK). Luciferase activity was quantified at 24 h using the dual-glo luciferase assay system (Promega, Southampton, UK), normalised using Renilla luciferase values from the same well and normalised values for control transfected cells (no pre-miR) were set to 1.0. Cell line identity was routinely confirmed using short tandem repeat analysis (Powerplex 16 System, Promega, Southampton, UK) and absence of mycoplasma was confirmed using the Mycoplasma PCR detection kit (Applied Biological Materials, Richmond, Canada).

## Results

### IGHV mutational status drive gene expression more strongly than BCR signalling capacity

mRNA sequencing achieved a mean of 7.13 × 10^7^ reads (range 4.37 × 10^7^–1.21 × 10^8^) per sample, of which 96.0% (range 92.4–98.8%) and 55.6% (range 42.2–64.2%) mapped to hg38 and Ensembl gene annotations, respectively (Fig. 1A). 15,529 genes passed filtering criteria (Fig. S2B). Exploratory PCA analysis delineated U-CLL-S, M-CLL-S and M-CLL-NS into three broadly distinct groups, with good separation of IGHV mutational status (Fig. 1B(i)).

Initially, we focused on the gene expression signature that differentiated M-CLL (regardless of sIgM signalling capacity) from U-CLL and identified 371 upregulated and 127 downregulated, differentially expressed genes (DEGs) at FDR < 0.05 (likelihood ratio test) and with a log_2_FC ≥ ±1 (Fig. 1B(ii)). Our data showed a high-level of concordance with previously published datasets when analysed quantitatively by overlaying differentially expressed genes from other studies onto our volcano plots and observing conservation of direction of differential expression (Fig. S5A), but also when directly comparing genes called as significant differentially expressed in other studies we were able to call 201/422 previously identified differentially expressed genes (Fig. S5B) [23–26]. We also were able to identify a significant panel of 297 novel DEGs, suggesting greater resolution in our study.

The upregulated DEGs (FDR ≤ 0.05, log_2_FC ≥ 1) in U-CLL included genes involved in RAS (*GNB4*, *GAB1*, *RASAL1*, *ANGPT2*, *FGFR1*, *IGFR1*, *INSR*, *KSR2*, *PLD1*, *ZAP70*, *PRKCA*), Wnt (*LRP5*, *VANGL1*, *VANGL2*, *WNT2B*, *WNT5B*, *WNT9A*, *FZD1*, *PRKCA*), and PI3K-Akt signalling (*GNB4*, *TCL1A*, *ANGPT2*, *CHAD*, *FGFR1*, *IGF1R*, *INSR*, *ITGB4*, *ITGB5*, *PRKCA*, *PPP2R3B*, *TNXB*) as well as other genes encoding proteins known to be over-expressed in U-CLL (*ZAP70*, *LPL*). Downregulated DEGs (FDR ≤ 0.05, log_2_FC ≤ −1) included those involved in MAPK (*DUSP2*, *DUSP8*, *NR4A1*, *TNF*, *MYC*), BCR (*CD86*, *CD40LG*, *EGR1*, *EGR2*, *EGR3*, *EGR4*), TLR (*TLR1*) and TNF signalling (*TNF*, *TNFRSF18*, *TNFRSF9*). Using the 498 DEGs in IPA and DAVID, we identified upregulated genes involved in Wnt signalling, down regulation of TNF and PTEN signalling genes, and increased expression of genes associated with cellular migration in U-CLL.

To probe the gene expression signature associated with BCR signalling capacity, we compared M-CLL-S with M-CLL-NS, excluding U-CLL. With thresholds at FDR ≤ 0.05 (likelihood ratio test) and log_2_FC ≥ ±1, the signature included 9 downregulated (*ITPRIPL2*, *TNFRSF9*, *CALHM2*, *KIR3DL2*, *MAF*, *IL1A*, *CSF1*, *FNBP1L* and *NT5E*), 0 upregulated DEGs in M-CLL-S (Fig. 1Biii) and no overrepresented pathways, although *MAF* is associated with B cell anergy in mouse models [27]. Whilst we anticipated detecting fewer DEGs between M-CLL-S and M-CLL-NS due to the reduced sample size, statistical power alone is unlikely to explain the pronounced difference compared to U-CLL vs. M-CLL and infers a limited impact of signalling capacity on the CLL transcriptome.

### miRNA expression strongly associates with IGHV mutational status

Small RNA sequencing achieved a mean of 1.15 × 10^7^ reads (range 7.02 × 10^6^–1.91 × 10^7^) per sample, of which 84.6% (range 74.1–92.0%) mapped to hg38 and 20.6% (range 7.4–40.2%) mapped to a mature miRBase 21 miRNA. Of the 2587 mature miRBase 21 miRNAs; 966, 699 and 922 were not detectable in any sample, filtered out due to low read counts and taken forward for analysis, respectively (Fig. S2C). Of our 25 most highly expressed miRNAs, 21/25 were observed in the top 50 most highly expressed miRNAs in a published dataset [28] and included key miRNA consistently reported as overexpressed in CLL vs. normal B cells (i.e. miR-150-5p, miR-155-5p, miR-146b-5p, miR-21-5p and miR-29a-3p [15, 29]), demonstrating the validity of our approach to extend on established findings.

The PCA of our miRNA data showed less prominent separation than our initial mRNA analysis. There was evidence of separation based on IGHV mutation status, but not based on BCR signalling subgroups in M-CLL patients (Fig. 1C(i)). Differentially expressed miRNA (DEM) analysis for IGHV mutation status identified 38 DEMs (16 downregulated and 22 upregulated in U-CLL) at FDR ≤ 0.05 and log_2_FC ≥ ±1 (Fig. 1C(ii)), but 0 DEMs for BCR signalling capacity (Fig. 1C(iii)). Comparison of our U-CLL vs. M-CLL miRNA expression signature with the key miRNAs characterised in CLL showed that 12 of the 17 miRNAs associated with IGHV status [30] behaved similarly in our study, and included the key CLL miRNAs miR-150-5p and miR-146a-5p, down- and upregulated in U-CLL, respectively [15, 31] (Fig. S5C). Of the miRNAs downregulated in U-CLL, miR-146b-3p and miR-146b-5p are downregulated in pancreatic cancer and aggressive lung cancer, respectively [32, 33]. Of those upregulated in U-CLL, miR-944, miR-138-5p and miR-338-3p are upregulated in advanced cervical cancer [34], over-expressed in bladder cancer [35] and thought to have tumour suppressor potential in lung cancer [36], respectively.

### 14q32 miRNA clusters is differentially expressed in IGHV subgroups

**Fig. 2:**
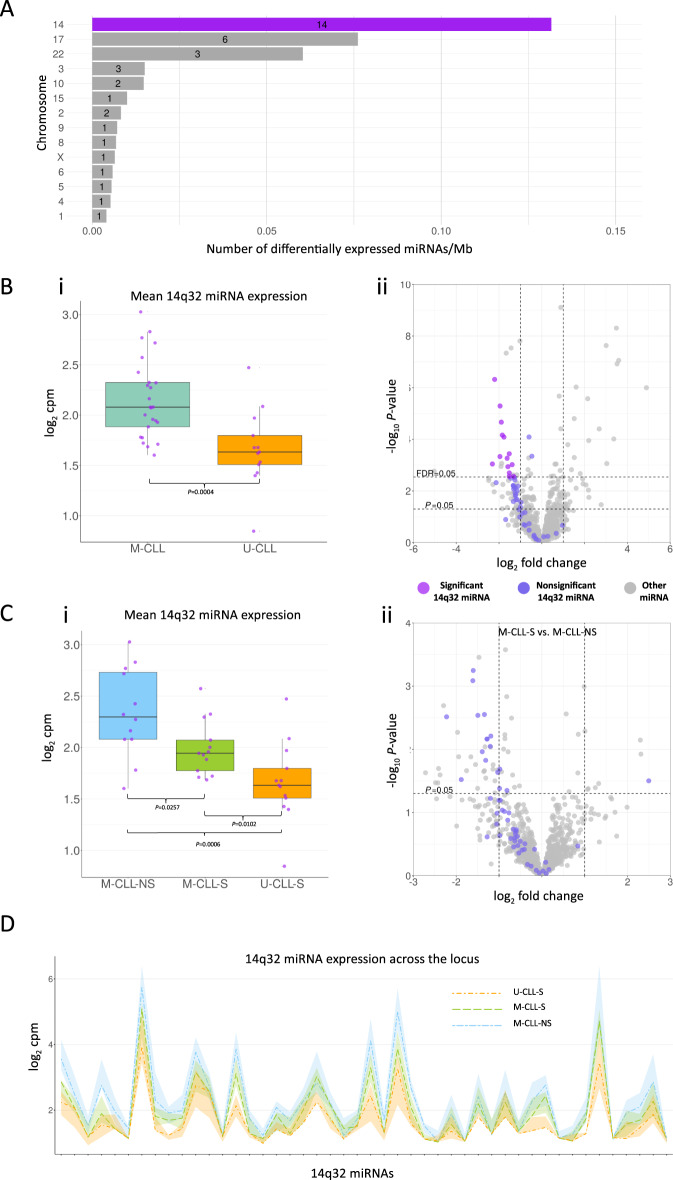
Identification of and expression of the 14q32 miRNA cluster in CLL. **A** Over a third of the differentially expressed U-CLL vs M-CLL miRNAs were located in 14q32 miRNA clusters. Number in bar indicates number of DE miRNAs per chromosome. **B** (i) Mean miRNA expression of the 14q32 locus is lower in U-CLL than in M-CLL. (ii) The majority of the 14q32 miRNAs (purple points) were downregulated in U-CLL compared to M-CLL. **C** (i) Mean miRNA expression of the 14q32 locus is lower in M-CLL-S than in M-CLL-NS and shown to decrease from M-CLL-NS to M-CLL-S to U-CLL-S. (ii) The majority of the 14q32 miRNAs (purple points) were downregulated in M-CLL-S compared to M-CLL-NS. **D** Ribbon plot showing the expression of each miRNA in the 14q32 locus ordered by genomic loci from 5′–3′. Dark lines indicate the group median for each miRNA, connection between points is for clarity and no relationship between adjacent miRNAs is inferred. Coloured ribbons indicate the interquartile range for each group. miRNAs plotted include only those expressed at sufficient level to be reliably detected (see “Methods”).

As miRNAs are often located in clusters, we noted that 37% of DEMs (14/38) mapped to two miRNA clusters in 14q32.2-q32.31, in a ~245 kbp locus known as the *DLK1-DIO3* imprinted region (chr14:100,825,000-101,070,000), approximately 4.5 Mbp upstream of *IGH* loci (Fig. 2A) where the lncRNA *MEG3* and downstream 14q32 miRNA clusters are transcribed from the maternal allele [37]. As only some of the 14q32 miRNAs were called as DEMs, we sought to understand expression of the 14q32 miRNA clusters in their entirety. The 14q32 clusters (*P* = 0.0004, Wilcoxon rank sum exact test) and 92.5% of the constituent miRNAs (49/53) were under-expressed in U-CLL compared to M-CLL (Fig. 2B) and the majority were downregulated in M-CLL-S compared to M-CLL-NS, albeit these differences did not reach significance at FDR ≤ 0.05 (Fig. 2C(ii)). Taken together, we observed high (log_2_cpm = 2.37), intermediate (log_2_cpm = 2.04) and low (log_2_cpm = 1.75) mean expression of 14q32 miRNAs in the M-CLL-NS, M-CLL-S and U-CLL-S subgroups, respectively (Fig. 2C(i)), and this was true for miRNAs across the locus (Fig. 2D). These observations were supported by unsupervised k-means clustering that showed enrichment of M-CLL-NS in high expression cluster (6/8), M-CLL-S in intermediate expression cluster (8/14) and U-CLL-S in the low expression cluster (10/16) (Fig. S6A). Some enrichment of DNA methylation epitypes [4] was observed in the k-means clusters however the relationship with 14q32 miRNA expression, IGHV mutation status and BCR signalling capacity was unclear (Fig. S6A, B).

**Fig. 3:**
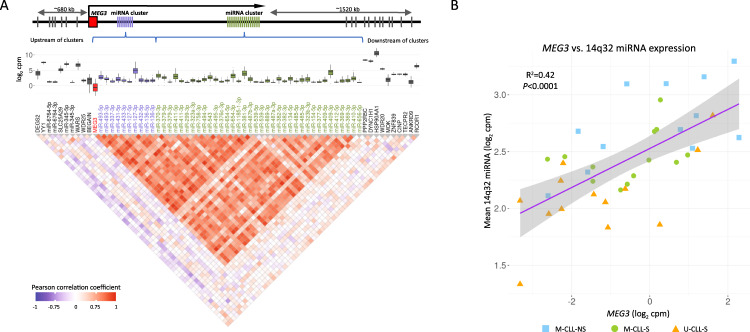
Co-expression of 14q32 locus miRNAs and mRNAs in CLL. **A** Correlation of expression of the 14q32 miRNAs and surrounding mRNAs/miRNAs depicted by a correlation plot. Red indicates a strong positive correlation between members of the 14q32 miRNA clusters and *MEG3*, blue a strong negative. miRNAs are plotted in the order that they appear in the genome, colour of miRNA name is used to separate miRNAs belonging to the 3′ and 5′ 14q32 miRNA clusters. Outside of the clusters (indicated by the genomic loci annotation) mRNA and miRNA levels do not correlate with the 14q32 miRNA cluster. Only miRNAs/mRNAs with sufficient read counts to be readily detected are depicted (see methods for inclusion criteria). **B** Correlation plot of mean 14q32 miRNA expression against *MEG3* expression.

Next, we investigated co-expression across the locus and demonstrated a strong positive correlation between expression amongst 14q32 miRNAs and with *MEG3*, but not with distal or proximal flanking genes/miRNAs outside of the 14q32 cluster (Fig. 3A), consistent with expression as a single, polycistronic transcript [38].

Then, we precluded confounding clinico-biological features on differential 14q32 miRNA expression. Expression of 14q32 miRNA in CLL was not associated with patient sex (Fig. S7) and whilst deletion of 14q is a rare but recurrent feature of the CLL genome, our copy number abnormalities (CNAs) analysis detected no deletions of 14q32 [39] (Fig. S8, Tables S1 and S2). We could not identify any impact of established chromosomal aberrations (del11q, +12, del13q and del17p) on 14q32 miRNA expression. Indeed, whilst our cohort was not designed to investigate these associations, any impact of recurrent cytogenetic lesions on 14q32 miRNA expression is likely to be minimal, given the almost ubiquitous presence of 13q14 deletions in our cohort (31/38 cases) and the paucity of cases with del17p (3/38), del11q (3/38) and tri12 (3/38) whose presence was not dissimilar in our three IGHV-BCR subgroups. Although the tumour purity of all cases was high (all samples ≥77% (Fig. S1C)), we performed confirmatory miRNA sequencing on purified CLL B cells and demonstrated a strong concordance with the non-purified dataset, suggesting that the 14q32 miRNA expression pattern was not driven by contaminating cells (Fig. S9A). Specifically, the 14q32 miRNAs followed the same pattern of being downregulated in U-CLL compared to M-CLL (Fig. S9B) and downregulated in M-CLL-S compared to M-CLL-NS (Fig. S9C) and we observed considerable overlap between the pre-/post-purification datasets.

Expression from maternal allele of the *DLK1*-*DIO3* locus is governed by the methylation states of two differentially methylated regions (DMRs) upstream of, and overlapping of the *MEG3* promoter (the *MEG3*-DMR and the IG-DMR respectively) [40]. Aberrant hypermethylation of the *MEG3*-DMR has been reported in various cancers [41]. However, in our data we did not observe significant differential methylation of the *MEG3* gene body, *MEG3* promoter, the *MEG3*-DMR or the IG-DMR (although the IG-DMR was poorly covered by the array) amongst our CLL subgroups, and we did not identify substantial variation in methylation levels in this locus amongst the cohort (Fig. S10).

### Expression of 14q32 miRNAs correlates with IGHV-dependant gene expression

Next, we aimed to identify biologically relevant miRNA:mRNA interactions, initially by analysing all DEGs (*n* = 498) and DEMs (*n* = 38). Pearson’s correlation was used to identify negatively correlated pairs of miRNA:mRNA which were intersected with databases of experimental and computationally predicted miRNA:mRNA interactions. The 198 significant, negatively correlated miRNA:mRNA pairs present in ≥1 database (in silico miRNA:mRNA interactions) were used to create a miRNA:mRNA interaction network for the IGHV mutational status transcriptional signatures (Fig. S11A). This network revealed multiple miRNAs as broad potential regulators of the CLL transcriptome, including miR-146b-5p, miR-543, miR-338-3p, miR-495-3p and miR-4476 targeting 38, 37, 15, 12 and 12 targets respectively (Fig. S11B). Several DEGs between U-CLL and M-CLL such as *TRIM2*, *TGFBR3* and *REPS2* were potentially regulated by four miRNAs each (Fig. S11C). Characterisation of the network mRNAs by KEGG pathway membership showed mRNAs involved in Wnt (e.g., *PRICKLE2*, *WNT2B*, *WNT5B*), MAPK (e.g., *DUSP2*, *MAPK4*) and Ras (*GAB1*, *FGFR1*, *PLD1*) and BCR signalling (e.g., *GAB1*, *EGR2*).

**Fig. 4:**
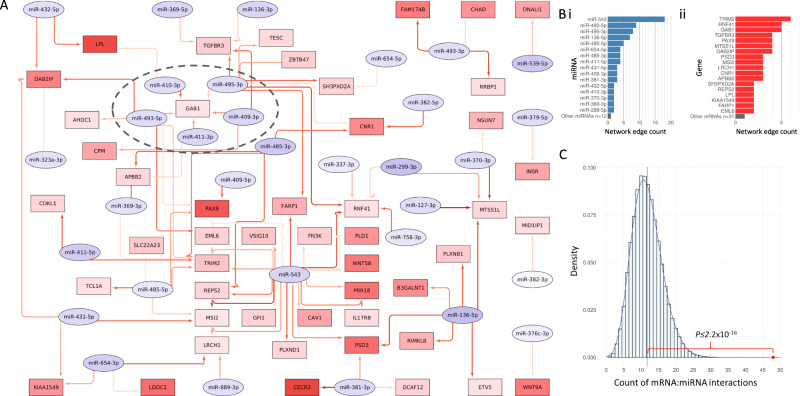
The potential impact of 14q32 miRNA on the U-CLL vs M-CLL signature. **A** A miRNA:mRNA interaction network showing the potential 14q32 miRNA regulation network of the top 200 U-CLL vs M-CLL mRNAs. Here, mRNAs are in rectangles, miRNAs are in ovals and are filled according to degree of under-expression (red) or over-expression (blue) in U-CLL vs. M-CLL. Red arrows indicate these features are negatively correlated and present in at least one putative or experimentally derived interaction database. Darker arrows indicate stronger negative correlations. *GAB1* and the network of regulatory miRNA that target it has been highlighted with a dotted oval. **B** Barplots showing the number of edges in the miRNA:mRNA interaction network for (i) each miRNA and (ii) each mRNA in the network. Features with one edge have been summarised for clarity (grey bar at bottom). **C** The number of edges in the 14q32 miRNA and top 200 U-CLL vs. M-CLL mRNA network is greater than expected by chance. We simulated 50,000 randomly selected, size matched miRNA:mRNA interactions present in miRTarget or miRDB (shown in histogram) and compared against the 48 miRTarget or miRDB edges seen in our network (red dot) using a one sample student’s *t*-test.

Twelve 14q32 miRNAs appeared in the network including miR-543, miR-495-3p, miR-409-3p and miR-411-3p. Next, we evaluated the potential impact of the 14q32 miRNAs on the strongest components of the IGHV-associated expression signature using the same network approach for the top 200 DEG (FDR ≤ 0.05, log_2_FC ≥ ±1 and ranked by *P* value) and all 53 of the expressed 14q32 miRNAs. The analysis generated a network of 90 interaction representing the 14q32 miRNA interaction network with the IGHV associated gene expression signature (Fig. 4A). Remarkably, our network analysis showed in silico interaction between the 14q32 miRNAs and 49 (24.5%) of the 200 mRNAs most strongly associated with *IGHV* mutation status. We found several core miRNAs with a strong potential impact on IGHV mutational status transcriptional signature (e.g. miR-543 was observed to have 18 mRNA interactions including *WNT5B* and *PLD1*) (Fig. 4Bi) and some mRNAs such as *TRIM2*, *RNF41*, *GAB1* and *TGFBR3* were potentially targeted by multiple miRNAs (6, 5, 5 and 4 miRNAs respectively) (Fig. 4Bii).

To test for enrichment of miRNA:mRNA interactions in our network, we tested 50,000 random samples of 200 mRNAs and 53 miRNAs and compared the number of putative interactions in the TargetScan and miRDB databases. We demonstrated that the number of interactions associated with our 14q32 network (48 interactions) was significantly greater than those observed in the randomly sampled, simulated networks (mean 11.75) (*P* ≤ *2*.2×10^-16^ from one sample Student’s *t*-test) (Fig. 4C).

### Several 14q32 miRNAs regulate *GAB1* mRNA and protein levels

**Fig. 5:**
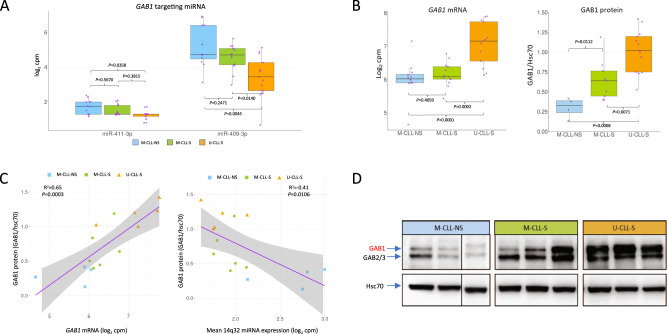
Expression of *GAB1* and *GAB1* targeting miRNA in CLL. **A** Boxplots of two miRNAs predicted to interact with *GAB1* in silico divided by CLL group. P-values calculated using Wilcoxon signed rank test. **B** Boxplots of *GAB1* mRNA and GAB1 protein expression for each CLL group. **C** Correlation of GAB1 protein with *GAB1* mRNA, and correlation of GAB1 protein with mean 14q32 miRNA expression. Correlation statistics generated using Pearson’s correlation coefficient. **D** Representative GAB1 immunoblots for protein level assessment.

**Fig. 6:**
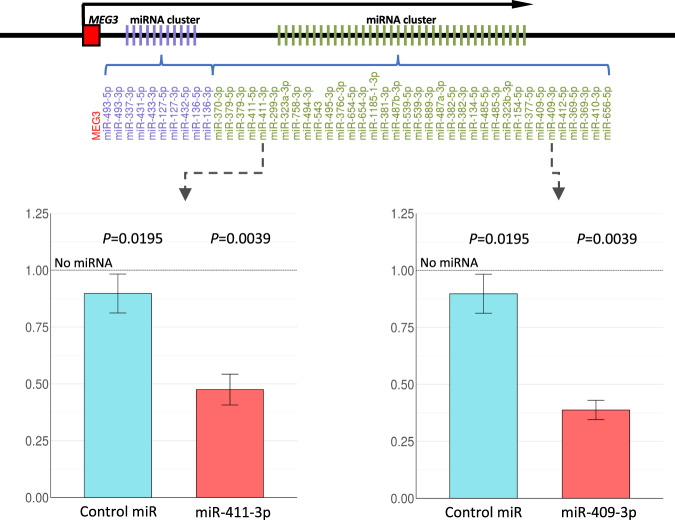
Relative luciferase intensity of luciferase expression vectors transfected alongside miRNA of interest or control miRNA. Relative luciferase expression ratios were calculated by dividing the control/test miRNA luciferase levels by a no miRNA control. Ratios were tested statistically using one sample Wilcoxon signed rank tests to mu of 1 (i.e., the no miRNA control). Error bars indicate standard deviation to mean. Upper annotation depicts the relative position of selected miRNA in the 14q32 miRNA clusters and relative to *MEG3*. Annotation is not to scale.

*GAB1*, which encodes a docking protein associated with increased BCR signalling in B cells [42], was a putative target of 5 miRNAs (miR-409-3p, miR-410-3p, miR-411-3p, miR-493-5p and miR-495-3p). These miRNAs were expressed in a similar manner as shown for the entire locus, i.e., high, intermediate and low mRNA expression in M-CLL-NS, M-CLL-S and U-CLL-S, respectively (Figs. 5A and S3B). Using immunoblotting, we confirmed that GAB1 protein levels mirrored mRNA levels in our patient subgroups (Figs. 5B, D and S3A). As such, both *GAB1* mRNA and protein levels were inversely associated with 14q32 miRNA expression levels (Figs. 5C and S3A). To confirm these interactions, we performed co-transfection experiments using pre-miR mimics and luciferase expression vectors with *GAB1* 3′-UTR in 293T cells. We chose two *GAB1* interacting miRNAs based on their location relative to *MEG3* and the level of support provided by the interaction prediction tools. The miRNAs miR-409-3p and miR-411-3p were able to repress activity of the *GAB1* 3′-UTR, with a 61% (*P* = 0.004, Wilcoxon sum rank test) and 52% (*P* = 0.004) reduction in reporter expression, respectively (Fig. 6). Cross reference against target prediction algorithms showed that miR-409-3p was a previously laboratory demonstrated *GAB1* targeting miRNA [43] whilst miR-411-3p and was predicted to interact with *GAB1* by miRTarget with a strong interaction score (85/100).

## Discussion

Understanding which CLL patients will progress requiring immediate treatment and which will have a more indolent course remains challenging. Previous studies have identified substantial differences in the mRNA and miRNA expression profiles of U-CLL and M-CLL and have linked these differences to the distinct clinical and biological features of these key disease subsets and regulation by signals in the tumour microenvironment [20, 21, 44, 45]. Whilst it is possible that these differences reflect the distinct cell of origin of these subsets, it is also plausible that they are impacted by the sIgM signalling capacity of the tumour cell that also varies between these CLL subsets, is associated with outcome [8] and may impact on the induction of anergy [46] which also results in a characteristic transcriptional profile [27].

To address this question, we employed high throughput molecular approaches to compare genome-wide mRNA and miRNA expression in three distinct subsets of samples defined by *IGHV* mutation and sIgM signalling status. Comparisons between signalling responsive U-CLL and M-CLL samples revealed the impact of cell of origin whereas comparisons between signalling responsive and non-responsive M-CLL samples were used to reveal the potential impact of BCR signalling capacity variation (within M-CLL). There are very few U-CLL cases without sIgM responsiveness so we did not include this rare subset in our analysis.

Our initial finding was that transcriptional variation of mRNA and miRNA in CLL appeared to be principally driven by IGHV-status, rather than sIgM signalling; we identified 371 upregulated mRNAs, 127 downregulated mRNAs, 22 upregulated miRNAs and 18 downregulated miRNAs between U-CLL and M-CLL, but only 9 downregulated mRNAs between signalling responsive and non-responsive M-CLL. The U-CLL vs. M-CLL DEGs/DEMs showed broad agreement with published studies [15, 30, 47], despite historical changes to miRNA nomenclature, technical differences and computational differences, and a degree of inconsistency between studies; for example, data is inconclusive on the relationship between miR-155-5p expression and IGHV mutation status [20, 21, 28]. In our study, miR-155-5p expression was not associated with IGHV status which may reflect the composition of our cohort as chromosomal aberrations linked to miR-155-5p expression such as trisomy 12 and deletion of 17p [31] were scarce in our cohort. The minor transcriptional differences between M-CLL-S and M-CLL-NS were surprising, given the association between signalling capacity and patient outcomes [8], however, this may reflect a cohort size that was insufficiently powered or factors such as tumour microenvironmental interaction, antigen driven/autonomous signalling, IGHV gene usage or somatic variants, may have limited our ability to detect in vivo BCR engagement induced anergic transcriptional signatures.

A striking discovery from the study was that 37% (14/38) of miRNAs that differed between U-CLL and M-CLL were derived from 14q32 miRNA clusters. Closer examination identified locus wide dysregulation of miRNA expression patterns both between U-CLL and M-CLL, and between M-CLL-S and M-CLL-NS albeit these differences were not significant. To rule out the contribution of known confounders we repeated the miRNA sequencing analysis on purified CLL cells and observed strong concordance with non-purified cell data, and we did not observe a strong association with patient sex. The inability of previous miRNA studies to identify dysregulation of the 14q32 locus between U-CLL and M-CLL is likely to be the result of the paucity of 14q32 miRNA coverage on microarray/qPCR platforms and the enrichment of the high 14q32 miRNA expression M-CLL-NS cases in our cohort.

Whilst our study is the first to report a role of the 14q32 cluster miRNA in CLL subtypes, the clusters have been extensively studied in solid tumours and other haematological neoplasms where a tumour suppressor role had been reported in melanoma (miR-376a/c), papillary thyroid (miR-654-3p), colorectal (miR-409-3p) and renal cell cancer (miR-411-5p) [43, 48–50]. In contrast, high-expression of 14q32 miRNA have been associated with inferior outcome in lung cancer and acute myeloid leukaemia, suggesting that the cluster may have cell-type specific functions [51, 52]. In splenic marginal zone lymphoma (SMZL) several 14q32 miRNA are down-regulated compared to normal B cells and CLL [53], suggesting the cluster may have a more expansive role in B-cell tumours. The strong correlation observed amongst individual 14q32 miRNA and the *MEG3* lncRNA supports mouse studies reporting expression from a ~200 kbp polycistronic transcript originating from the *MEG3* TSS [38]. *MEG3* was the first tumour suppressor lncRNA identified, has been demonstrated to have a pathogenic role in a number of cancer models [54, 55], and is down-regulated in a number of primary tumours in comparison to matched normal tissues [56, 57]. In our study, the strong co-expression of *MEG3* and the 14q32 miRNA cluster precluded the possibility of studying these *loci* independently.

*MEG3* and the 14q32 miRNA are located within the *DLK1-DIO3* genomic imprinted region, controlled by the paternal/maternal allele specific methylation of the two differentially methylated regions (DMR) in normal cells, the IG-DMR 13 kbp upstream of the promoter and the *MEG3*-DMR overlapping the *MEG3* promoter [40]. Aberrant DMR methylation is observed in several leukaemias, including hypermethylation of the *MEG3*-DMR in AML, MDS and myeloma [41, 58], and loss of IG-DMR imprinting is associated with increased *MEG3* and 14q32 miRNA expression in acute promyelocytic leukaemia [59]. In our CLL cohort, we did not identify differential DMR methylation. However, the arrays used had limited coverage of the IG-DMR, compromising our analysis and emphasising the need for analysis of this cluster at greater resolution.

In addition to differential expression of the 14q32 locus, we observed a stepwise increase from U-CLL-S to M-CLL-S and M-CLL-NS, that was apparent in both purified and non-purified samples. High BCR signalling capacity has been demonstrated to be a poor prognostic indictor, independent of IGHV mutation status [8]. As such, the potentially inverse association between 14q32 miRNA expression and prognosis in our CLL subgroups is notable in that it would coincide with the tumour suppressor potential of the 14q32 miRNA and *MEG3* lncRNA. Our study was not designed to discriminate survival differences associated with 14q32 miRNA expression independently of other factors, but it would represent an area of exciting potential for future work.

When we classified our patients by DNA methylation epitype we showed the expected enrichment of m-CLL and n-CLL in the M-CLL and U-CLL subgroups. The analysis of a larger CLL cohort would be needed to establish the exact relationship between epitype, IGHV status, BCR signalling competence and 14q32 miRNA expression, but our preliminary analysis did suggest that 14q32 expression is more associated with IGHV and BCR signalling than epitype.

Remarkably, our miRNA:mRNA interaction network analysis showed that the 14q32 miRNA have potential for broad perturbation of entire networks of miRNA-mRNA interactions, where 49 (24.5%) of the 200 strongest features of the IGHV associated transcriptional signature are potential targets for 14q32 miRNAs which was significantly enriched compared to randomly sampled miRNA:mRNA. Experimental limitations include our computational approach, which whilst typical for these studies does assume some functional interaction to infer negatively correlated mRNA and miRNA, and the fact that miRNA levels are not the only determinant of mRNA abundance. Despite these limitation, our network analysis implicates 14q32 miRNAs as significant determinants of differences in mRNA expression between U-CLL and M-CLL.

Although our miRNA:mRNA network was constructed with in silico predictions, we identified interactions of known biological significance. For example, miR-409-3p is known to regulate *GAB1* in colorectal cancer [43], and we were able to further confirm the mechanistic link between miR-409-3p and another miRNA, miR-411-3p, with the regulation of *GAB1* in our models. Therefore, in addition to the previously reported ability of miR-150-5p to reduce *GAB1* expression in CLL cells [21], we now show that two further miRNA, miR-411-3p and miR-409-3p, located at 14q32 also regulate *GAB1* and associate with IGHV and BCR signalling status. In cytokine signalling, GAB1 is thought to act as an adaptor molecule to transmit signals to ERK MAP kinase [60]. Upon receptor activation GAB1 becomes tyrosine phosphorylated and associates with a number of different proteins including Pi3K which are key adaptor molecules for BCR signalling [61]. Specifically in CLL, *GAB1* is expressed at greater levels in patients with higher BCR signalling capacity and is consequently associated with a worse clinical outcome [21]. Upon BCR engagement, a number of different Protein Tyrosine Kinases such as SYK, LYN and FYN phosphorylate membrane recruited GAB1 [42, 62, 63], leading to activation of Pi3K and pro-survival signalling factors [45]. Additionally, GAB1 also acts as docking/scaffolding for BCR signalling components PLCγ, CRK and CRKL proteins [64]. Consequently, down modulating *GAB1* expression may result in impaired BCR signalling.

Questions remain about the ability of 14q32 miRNA to regulate the expression of *GAB1* in vivo and their role in other B-cell malignancies, during B-cell development, amongst cytogenetic subgroups and in response to treatment, all of which represent promising opportunities for future work. Herein we suggest that multiple miRNAs within the 14q32 clusters can target *GAB1* in CLL and may explain the difference in BCR signalling capacity and we speculate that the dysregulation of multiple miRNAs could provide precise regulatory control, reduce the degree of redundancy in the epigenetic regulation of key mRNAs, or have an additive impact on transcriptional regulation but these hypotheses remain to be confirmed. Whilst the association between *GAB1* and 14q32 miRNAs was evident based on IGHV status, the same association failed to reach statistical significance in the M-CLL cases with differing BCR competence, that may, to some degree, reflect suboptimal statistical power that would be overcome with future screening of a larger cohort of CLL patients. Our data hint at an association between 14q32 miRNA expression level and survival that may suggest value for 14q32 miRNA quantification as prognostic biomarkers. Further investigation and validation of the methylation dependent regulation of the 14q32 locus in CLL, perhaps using long read technologies that offer high resolution DNA sequencing, methylation status and phasing of methylated CpGs across the locus would highlight the potential for demethylating agents to relieve repression of 14q32 miRNA and *MEG3* in high risk CLL subsets.

Our data illustrates the critical role of miRNA-mediated regulation in the pathobiology of CLL, showing that the 14q32 miRNA have a putative regulatory role in IGHV-associated transcription, with functional evidence of a mechanistic interaction with *GAB1*. Given that these naturally produced molecules and their levels can be readily regulated with miRNA mimics or miRNA/antagomiRs, their therapeutic manipulation may have implications for more effective deployment of existing targeted agents.

## Supplementary information

Supplementary Methods

Supplementary Figures

Supplementary Tables S1 and S2

## Data Availability

mRNA and miRNA sequencing data and DNA methylation array data are available at ArrayExpress (https://www.ebi.ac.uk/arrayexpress/) under accession numbers E-MTAB-12017, E-MTAB-12023 and E-MTAB-12018 respectively.
